# Two Challenges to “Embodied Cognition” Research And How to Overcome Them

**DOI:** 10.5334/joc.151

**Published:** 2021-02-16

**Authors:** Rolf A. Zwaan

**Affiliations:** 1 Department of Psychology, Erasmus University Rotterdam, 3000 DR Rotterdam, The Netherlands

**Keywords:** embodied cognition, replication, open science, team science, language processing

## Abstract

From the time the notion “embodied cognition” has entered the field, researchers have been concerned about its meaning. Does the term refer to a coherent theoretical framework? Despite these concerns, use of the term “embodied cognition” has increased over the years to plateau in recent years. I will argue that the best way forward is not to search for evidence for or against some vague label but rather to systematically, in large-scale projects, address a series of questions that focus on well-defined cognitive tasks. Such projects ought involve preregistration, replication, and open materials, code, and data. For this enterprise to take off, it is important that incentives in the field be aligned with the goal to increase the reliability and validity of our research. There is reason to be optimistic that such an alignment will occur in the near future.

The notion of “embodied cognition” has become commonplace in psychology over the past two decades. ***Figure 1*** is an informal analysis of the use of the term “embodied cognition” in the scientific literature during this period. The search was performed in the Scopus and Web of Science databases for the period bookended by the years 2001 and 2020. The Scopus search was restricted to the title, abstracts, and keywords of a given paper with the search string PUBYEAR > 2000 TITLE-ABS-KEY (“embodied cognition”). In Web of Science I used the following settings: **ALL FIELDS**: (“embodied cognition”) *AND* **DOCUMENT TYPES**: (Article) *Indexes=SCI-EXPANDED, SSCI, A&HCI, CPCI-S, CPCI-SSH, ESCI Timespan=2001–2020*).

**Figure 1:**
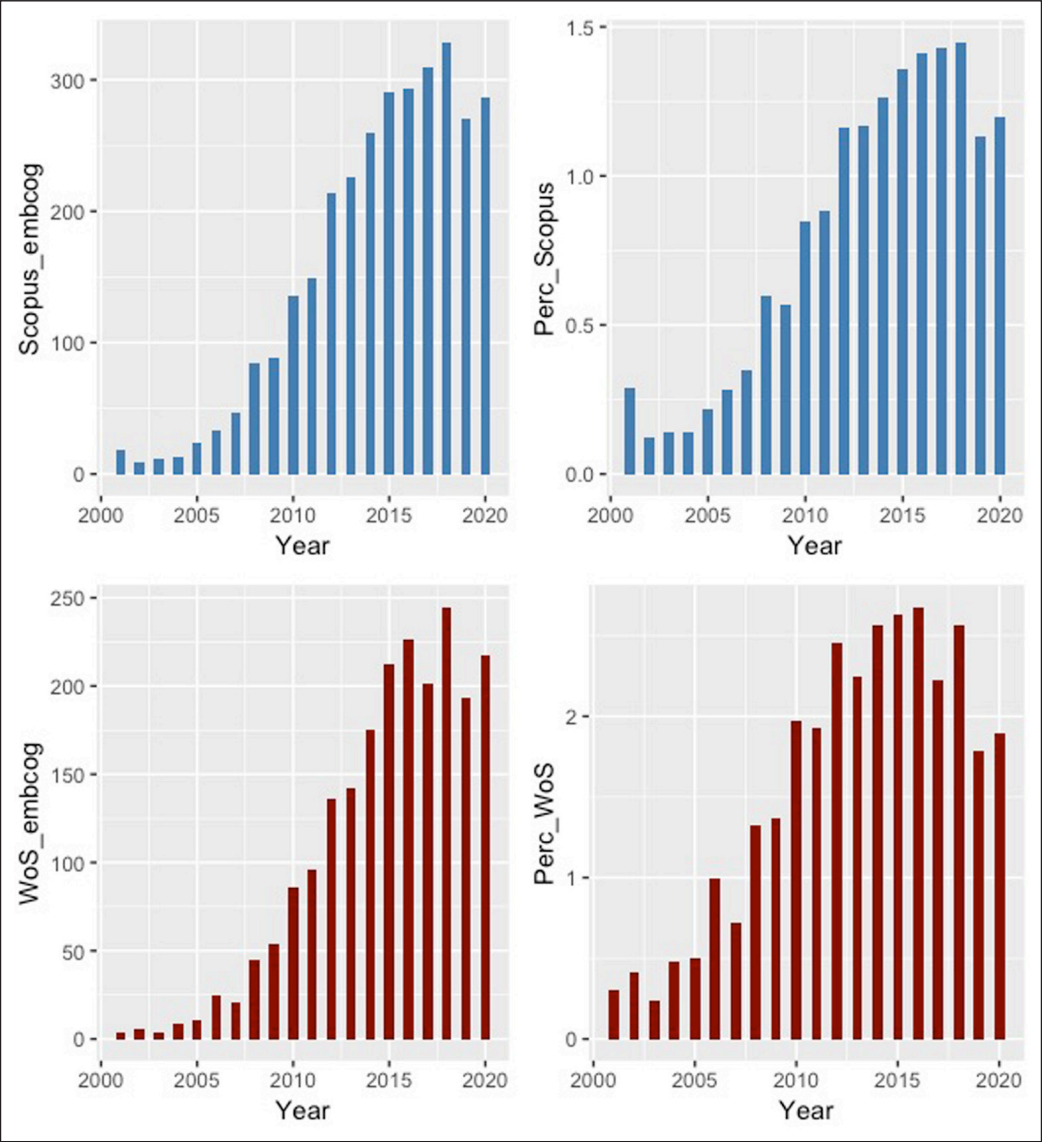
Number of mentions of “embodied cognition” per year between 2001 and 2020 in Scopus and Web of Science (left-hand panel) and percentage of mentions “embodied cognition” of total number of mentions of “cognition” in Scopus and Web of Science during the same period (right-hand panel). The code and data to reproduce this figure can be found at: *https://osf.io/d9az8/?view_only=bcb47f128adc4d35baa19a81d2419a5d*.

This analysis was performed on January 29, 2021. The left-hand panels show a very rapid increase in the number of mentions of “embodied cognition” and a leveling off in recent years.

An obvious concern about this analysis is that it does not correct for the growth of the literature in general. If the literature grows, then it stands to reason that the number of papers on embodied cognition grows as well. The right-hand panels reflect an attempt to correct for the growth of the literature by calculating the percentage of “embodied cognition” papers out of the total number of “cognition” papers (i.e., papers having “cognition” in the title, abstract, or keywords). Again, an initial increase in the number of “embodied cognition” papers is shown, followed by a leveling off in recent years.

There are a few other caveats to this analysis. First, the records for 2020 are likely not complete yet, which means the actual number of hits for that year might be higher than what is represented in the figure, which would primarily be reflected in the absolute number of mentions in the left-hand panels. Second, the analysis only uses the search term “embodied cognition” and therefore misses studies on the topic that do not use this precise term. Third, as “embodied cognition” becomes more common, it might become less useful to certain researchers, as it has become less distinctive. Fourth, it could be that use of the term “embodied cognition” has spread from some areas to others, for example from more fundamental fields (e.g., cognitive psychology) to more applied ones (e.g., educational psychology, marketing). A more fine-grained bibliometric analysis might uncover such a distribution over time.

These caveats notwithstanding, there are as yet no (bibliometric) signs the interest in “embodied cognition” is waning. This makes it all the more relevant to address challenges facing research that occurs under the rubric of “embodied cognition.” Recently, Ostarek and Huettig (2019) discussed six challenges to embodied cognition research. In this article, I will focus on two major challenges to “embodied cognition,” the *definitional challenge* and the *methodological challenge*, the latter of which overlaps with one of the challenges discussed by Ostarek and Huettig, although their focus differs from mine. I also propose ways in which these challenges can be met and point out that there are reasons to be optimistic about the success of this endeavor.

## The Definitional Challenge

Even though I used “embodied cognition” as part of a search string, it is legitimate to wonder what is actually meant by “embodied cognition.” One might even wonder, what is “embodied” and what is “cognition”? Under the rubric of embodied cognition, we can find research inspired by theories that originated in the 1980s and 1990s, such as linguistic theories about the role of metaphors in language and cognition (Lakoff and Johnson, 1980), neuroscientific theories about grasping behavior and action and language understanding (Rizzolatti & Arbib, 1998), as well as a more conventional cognitive psychological theory about mental representations in language comprehension, memory, and thinking (Barsalou, 1999). Among the empirical studies of embodied cognition, one can find experiments on grasping, lexical priming, learning, emotion, action understanding, discourse comprehension, education, social cognition, and marketing, just to give a non-exhaustive listing of topics.

It is not my goal here to review these theories or the associated empirical studies. I merely want to point out that when one looks under the hood of “embodied cognition,” what one finds is not a purring scientific engine. Rather, what unfolds itself before our eyes is a large collection of parts, many of which do not seem to be interconnected, and a good many of which may not even belong in the vehicle in question. That “embodied cognition” is anything but a coherent field, is not an original observation. An early review article on the topic is titled “Six views of embodied cognition” (Wilson, 2002). The author makes the point that not all of these six views are compatible or even truly “embodied.” A few years later, an article appeared called “Constraining Theories of Embodied Cognition” (Markman & Brendel, 2005). Apparently, the field had not yet succeeded in unifying the different approaches that operated under the banner of embodied cognition. And a decade after this, an article appeared entitled “Embodiment and language comprehension: reframing the discussion” (Zwaan, 2014), in which the more general concern was expressed that the discussion about embodiment had reached an impasse. Even though this last article has a narrower focus than the other two, as it only focuses on language comprehension, it illustrative of the same concern about the lack of a coherent research program, which goes to show that this concern, which is also the motivation behind this special issue, has been a constant factor. And, to be fair, the lack of theoretical coherence is not unique to “embodied cognition”; one can find many similar examples in the field of psychology. Nevertheless, the current special issue is a worthwhile effort to change the status quo with regard to “embodied cognition.”

To bring about such a change, it is useful to first consider how we arrived at this situation. Setting aside intra-scientific issues for the moment, we must consider what I will call para-scientific issues. A key para-scientific issue is that there is systemic pressure on researchers to frame their research in the most general terms. This is true for communications to grant review panels, in which researchers are told they have to make a convincing case to a panel with a broad set of interests, or even to society at large, and scientific journals, which ask authors to provide “highlights” of their articles. It is also true for communications to university press offices or the press itself, which cares mostly about attracting readers or at least clicks. And of course it is also true for authors who wish to publish in the most prestigious (though not necessarily the most rigorous) journals. They know that, to avoid the dreaded editorial buzz kill that “your article is more suited to a specialty journal,” they have to frame their studies in the broadest manner possible. This systemic pressure might explain why the term “embodied cognition” is featured so prominently in many articles that do not seem to have a common theoretical framework and why as a search term it still retrieves a considerable number of research articles each year.

It is perhaps no exaggeration to state that the field of cognitive psychology has made the most progress when researchers study relatively well-specified tasks, such as visual search, spoken word recognition recognition, anaphoric resolution, episodic memory retrieval, syllogistic reasoning, mental arithmetic, text recall, and so on. Embodied cognition is not a task, nor can it even be considered to be a field. It is best thought of as a diverse collection of studies and theories in various fields on which the same label is slapped.

I will limit myself to the field of language processing here, for two reasons. First, it is the area I am the most familiar with. Second, if even in this subdomain of cognitive psychology there does exist a unified theoretical framework of “embodied language processing,” this does not bode well for the field of cognition in general. As such, it is a useful testcase.

Some studies in embodied language processing are motivated by conceptual metaphor theory, others by perceptual symbol theory, and yet others under mirror neuron theory, just to focus on the most prominent ones. It does, therefore, not make sense to ask: “is language processing embodied?” in a general sense. After all, what is meant by “embodied” and, now that we’re asking, what is meant by “language processing”? Does a finding that applies to words also apply to sentences and connected text? And can we extrapolate from a lab experiment across communicative situations? I wrote about communicative situations and the degree to which they are embedded in the environment and how this might have implications for the use of perceptual symbols before (Zwaan, 2014) and will focus here on the level of analysis instead.

A key question concerning the level of linguistic analysis is: to what extent can findings obtained with regard to one level of analysis be extrapolated to other levels? In what follows, I only provide abstract examples. I am fully aware that there is a vast literature that may be or appear to be relevant to the issues I am addressing but, as I already mentioned, this article is not intended as a review of that literature and mentioning specific findings would only detract from the points I am trying to make.

Suppose one obtains evidence that the visual presentation of action words is reliably associated with corresponding brain activation (e.g., pinch verbs interfere with pinching actions, and/or vice versa, grasp verbs with grasping actions, and so on) of the motor system. Suppose even that studies find that processing the word is impeded by concurrent activation for the (pre)motor cortex (via concurrent manual actions or via transcranial magnetic stimulation). Suppose further that item analyses show the effect generalizes across verbs. Can we now safely conclude that motor activation occurs during language processing?

We cannot. The first obvious objection is that we cannot generalize across languages from a study in a single language, but my focus here is on the simple fact that words rarely occur in isolation (although solitary words are common in psychology experiments). Thus, the role of the motor system could be an artifact of the fact that the word is presented without context. Perhaps participants, upon reading or hearing the word, interpret it as a command: “pinch” which then primes them for the action.

Furthermore, even if (1) such action verbs were shown to reliably involve the motor system across contexts and languages and (2) inhibition or stimulation of areas in the motor system corresponding to the actions denoted by the verbs affects the processing of these verbs, then it is still difficult to maintain that the motor system is centrally involved in language comprehension. Suppose we are interested in the comprehension of simple narratives, a reasonably well-specified real-world task on which there exists a great deal of research. If one considers the distribution of simple action verbs in a corpus of narratives or expository texts, one would find that they are rarely of central importance to the story. Most texts are not about simple actions such as picking and kicking. Even children’s stories are about more abstract topics. “The Ugly Little Duckling,” for example, is about social exclusion and personal transformation rather than about swimming, quacking, and flapping one’s wings.

The text genre for which simple actions are the most germane are procedural texts such as recipes (e.g., *stir, knead, chop, whisk*) or manuals for household implements. It is difficult to envision what motor action would be relevant to slightly more complex actions that are described in a such as ordering a beer, paying a bill, or parking a car, let alone such complex actions as building a house, driving to Spain, or baking a cake, or abstract actions such as developing a theory or pondering the meaning of life (see also Zwaan, 2014). This is by no means meant to discourage researchers from studying motor activation in narratives. After all, it has been found in an educational context that having children enact actions from a story can enhance their reading comprehension (Glenberg et al., 2004). Rather, my point is that a task analysis would suggest that it is not likely to play a central role in narrative comprehension but that it might be in the comprehension of other text types and it is therefore important to consider task and genre constraints in studies of “embodied cognition” and language.

To take a different tack, again suppose a single-word study that compares the activation of lexical and visual representations upon visual word presentation and finds that the former are activated first. Can we then safely assume that the same occurs during narrative comprehension? For example after reading hundreds of pages of *The Lord of the Rings* in which an entire narrative world has been constructed, would a reader still first activate lexical associations to “Gandalf” or is it possible that visual representations now take precedence? After all, if a visual representation has been built up incrementally when reading various descriptions in prior parts of the novel, this representation might be easily accessible during subsequent comprehension and therefore be activated more quickly than one would surmise from single-word experiments. Whether or not this is the case is an interesting empirical question. Again, one cannot extrapolate from one linguistic unit to another.

The definitional challenge can be reduced to one of properly defining the scope of one’s study. As researchers we need to be much more specific about the limitations of our findings. We should first consider the level of analysis (words, sentences, texts), the type or genre (e.g., nouns, declarative sentences, simple narratives), the language (e.g., English, Spanish, Chinese) and the task at hand and use these to define as best as possible the constraints on generalizability of our findings (Simons, Shoda, & Lindsay, 2017). To be sure, this makes the projected impact of our work a great deal more modest, but it is also much more workable if the ultimate goal is to create a coherent theory of cognition. For this to happen, it is important to address the methodological challenge, which I will discuss next, as well as systemic impediments, which I will discuss in the final section of this article.

## The Methodological Challenge

The second challenge I want to address concerns methodology, and more specifically the reliability of results and the validity of measures. So far in this article I have conveniently assumed that all of the findings that have been published under the rubric of “embodied cognition” are reliable and valid. Reality is different, as it no doubt is for most areas in psychology and surrounding fields.

Let us first consider reliability, which is the likelihood that a measure consistently produces similar results. It has become clear in recent years that psychology has a replicability problem (e.g., Open Science Collaboration, 2015). Although this problem is perhaps larger in some areas of the field than in others, it is important to recognize that is it important to have a body of replicable findings for whichever research question one is interested in (e.g., Pashler & Harris, 2014; Simons, 2014; Zwaan, Etz, Lucas, & Donnellan, 2018).

Perhaps the most-cited empirical cognitive psychological study that falls under the rubric of “embodied cognition” is Glenberg & Kaschak (2002). This study found support for the action-sentence compatibility effect (ACE). In that experiment, participants judged the sensibility of sentences such as “Mike gave Art the pizza” by moving their hand to press a button. To do so, they had to move their hand from a central button to a button away from or toward their own body. The results indicated faster responses when the action performed by the participant was congruent with that of the sentence protagonist (e.g., away from the body in the case of the example sentence) than when it was incongruent (toward the body).

A large group of researchers, admirably spearheaded in part by the authors of the original article, recently performed a direct replication of these findings in a registered report (Morey et al., in press). None of the 18 participating labs was able to replicate the ACE. Does this set of findings count as evidence against “embodied cognition”? As the discussion in the previous section has shown, the answer to this question is a resounding no. Does the massive null result then count in a more circumscribed way as evidence against the role of the motor system in sentence comprehension? It certainly does not support this claim but the claim is too broad to be dismissed by this very narrow set of null results. Does the finding count as evidence against the ACE, then? It certainly counts as evidence against the ACE using the paradigm used in the original study.

And here we have run up against a limitation of direct replications. One cannot make inferences beyond the paradigm that is being used. When an original finding is replicated, one can say that one has a procedure in hand that reliably produces an effect, which is crucially important (Zwaan et al., 2018). Similarly when an original finding is not replicated (even when it is not replicated 18 times) one cannot make inference beyond the paradigm that is being used. Therefore, a direct replication is a good start but has its limitations. The logical next step is to turn to validity.

A measure is valid if the results obtained with it reflect the construct under consideration. Even if a finding is highly reliable, there may still exist questions concerning its validity. It could be that, yes, a procedure reliably produces an effect but that effect is an artifact of the specific method being used; it does not reflect the construct under study or does not do so to a sufficient degree. Suppose, for example, that someone would claim that there is a better way to test the ACE than was done in the original study. Researchers might, for example, claim the task is too indirect and therefore too noisy to find effects. Or researchers might claim that the effect is too short-lived to manifest in a post-comprehension task, which the ACE task might be considered to be.

The ACE replication project could fairly be criticized by the observation that it was hamstrung in that all participating labs used the exact same task and stimuli (see Yarkoni, in press for a more far-reaching criticism of large-scale replication projects). If the task is the wrong tool for the job, so the argument would go, then no wonder that nobody found anything. Of course, this criticism leaves unaddressed why the original study (or, in fact, several studies) found the effect in the first place but the the crucial point here is that direct replications are not the proper tool to test a broader hypothesis.

To test the more general hypothesis that sentence comprehension involves (in some way) motor activation, a different approach is needed. It requires the use of extensions to the method, which are often called conceptual replications. Such replications would involve a variation on the original experiment. It would, for example, involve an extension to a different task (e.g., naming vs. lexical decision), to different parameters (e.g., an SOA of 500 ms instead of 200 ms.), to a different subject population, to a different set of stimuli, and so on, conceptual rather than direct repplications, in other words.

Why not perform exclusively conceptual replications then? Detractors of direct replications have argued for precisely this (e.g., Stroebe & Strack, 2014). However, the problem with conceptual replications is that they are biased against the null hypothesis (Pashler & Harris, 2012). If one obtains a null effect, researchers could always talk themselves into believing that the conceptual replication was not a proper test of the hypothesis after all. They could then decide to run other conceptual replications. If one performs a sufficient number of conceptual replications, then a number of them are likely to show the effect by chance. If only the significant conceptual replications are published, which is the phenomenon of publication bias, is it is easy to arrive at a set that is likely to be mistaken for a mighty empirical edifice in support of the hypothesis.

The problem with this reasoning is that none or few of the conceptual replications might survive a direct replication (Simons, 2014; Zwaan et al., 2018). Does this mean then that extensions are useless? Quite the contrary. In an ideal world one would use a combination of direct and conceptual replications, so that one can in one fell swoop assess the reliability of a set of procedures, all intended to test the same hypothesis. The interest in large-scale collaborations seems to have increased over the past years, as quite a few have been published in recent years. Moreover, an infrastructure exists that facilitates such collaborations, namely the Psychological Science Accelerator (Monshontz et al., 2018).

A recent attempt in this direction crowdsourced ways to test a set of existing hypotheses and then performed meta-analyses across the different tests (Landy et al., 2020). This is a promising approach. The different ways to test a hypothesis could be regarded as conceptual replications. The approach used by Landy et al. avoids publication bias in that the participating studies were each preregistered. Moreover, each operationalization was developed independely and meta-analyses were performed not only to assess support for the hypothesis in question, but also to examine which of the empirical results are contingent on the decisions scientists make as they design their study.

Consider again the question of whether sentence comprehension involves motor activation. Admittedly, the question might need further specification but it suffices for our current expository purposes. A group of labs interested in testing the idea (a mix of proponents, neutrals, and sceptics would be ideal) would assemble. Following the approach of Landy et al. (2020), each lab would produce its own operationalization of the research question. All experiments would be preregistered. It might be possible to group the operationalizations according to certain criteria (e.g., how implicit each measure is). The participating labs would subsequently conduct their own experiment as well as one or more direct replications of an experiment that was designed by one of the other labs. This procedure will result in a range of tests of the hypothesis designed according to the field’s best collective understanding of the hypothesis in question with at least one direct replication per experiment. It might furthermore be possible to meaningfully categorize the tests in groups.

Several outcomes are possible. At one extreme, all experiments show the predicted effect. This would strongly support the hypothesis. There is a set of paradigms that reliably show the effect. At the other extreme, none of the experiments shows an effect. This would count as very strong evidence against the hypothesis and might be sufficient reason to abandon it altogether. All other outcomes would fall somewhere in the middle. An interesting case would be if a certain category of operationalizations shows the effect while another does not, as this might give rise to further theoretical and empirical work.

## Conclusions

I started out with a small bibliometric analysis, which suggests that the popularity of the term “embodied cognition,” after an initial sharp rise seems to have leveled off and thus is not on the wane. I noted that this makes it all the more important to address two major challenges that research on embodied cognition faces and I proposed ways to do so. The first challenge is the definitional challenge, which has bedeviled the field from the first time the phrase occurred in the literature (in both Scopus and the Web of Science, this is the year 1996). I have argued that there exists systemic pressure on researchers to define their study as broadly as possible but that this works against the need for a precise framing of the scope of the study and the implications of the results. I have pointed to greater precision about the theoretical framework and specifying the constraints on generalizability of the study as ways to overcome this challenge.

The second challenge concerns questions about the reliability and generalizability of studies. These questions can and ought to be raised about any area in psychology. Therefore, they are not meant as a criticisms of specific research projects. Rather they reflect an attempt to show how research can benefit from new developments in the field, such as open science, preregistration, and team science.

These conclusions point to the biggest challenge of all: to change the cognitive biases and incentives that drive the field. As Munafò, Chambers, Collins, Fortunato, and Macleod (2020) recently noted, the strong motivation that researchers feel to produce interesting results, is a double-edged sword, as this motivation makes them subject to apophenia (seeing patterns in noise) and to confirmation bias. At the same time, there is, as I mentioned earlier, systemic pressure to produce research that can be published in “high-impact” journals.

Can these incentives be changed? There are reasons to be optimistic (Munafò et al., 2020). For example, many journals now offer the opportunity to submit or even require preregistrations, which reduces threats to reliability because it reduces researcher the effects of cognitive biases. For example, by stating beforehand the outlier criteria prevents strategically amending these after the results are known. By stating the predictions beforehand, one prevents oneself from HARKing (Hypothesizing After the Results are Known, Kerr, 1998).

Also important is the push toward transparency in research methods and the use of data repositories, which seems to be gaining strength (e.g., Morey et al., 2016). Several countries, such as the United Kingdom and the Netherlands, have developed researcher-driven networks for open science (see for example *https://openscience-utrecht.com/code-of-conduct/*). To bring about such fundamental changes, it is important that the scientific community finds ways to best conduct team science such that each contributor to a project receives the credit they deserve (e.g., Bennett & Gadlin, 2012).

Another relevant development is the movement toward team science, as most clearly exeplified by the Psychological Science Accelerator (Monshontz et al., 2018). In fact, a team science approach could be targeted at different stages of the research process. For example, researchers in a specific domain could crowdsource the most relevant original hypotheses to test or existing findings to replicate, although more princpled ways to arrive at the latter have also been proposed (e.g., Field, Hoekstra, Bringmann, & van Ravenzwaaij 2019; Isager et al., 2020). I already discussed the project by Landy and colleagues (2020) who crowdsourced study designs to test a set of hypotheses. Another team-based project focused at the data-analytic stage of the research process and crowdsourced different analytic approaches (Silberzahn et al., 2018). Collectively, these efforts show that in might be fruitful to adopt a team science perspective could be applied to different phases of the resesearch process. Such aproaches help the field overcome or at least make transparent unavoidable researcher biases as well as limitations with regard to the generalizability of results.

I started out with a bibliometric analysis of “embodied cognition.” If the large-scale and smaller scale changes that I have described and proposed here take place, it is likely that a bibliometric analysis using that search term conducted one or two decades from now will show decreasing numbers of studies over the years. Far more importantly, we may at that time have obtained a far better understanding of the role of the body in specific cognitive tasks than we currently do.
